# Type material of Platyhelminthes housed in the Helminthological Collection of the Oswaldo Cruz Institute/ FIOCRUZ (CHIOC), Rio de Janeiro, Brazil, from 1979 to 2016 (Rhabditophora, Trematoda and Cestoda)

**DOI:** 10.3897/zookeys.662.11685

**Published:** 2017-03-20

**Authors:** Daniela A. Lopes, Adriana Mainenti, Marcelo Knoff, Delir Corrêa Gomes

**Affiliations:** 1 Laboratório de Helmintos Parasitos de Vertebrados, Instituto Oswaldo Cruz, FIOCRUZ, Av. Brasil, 4365 Rio de Janeiro, RJ, Brazil

**Keywords:** Catalogue, cestodes, holotype, paratype, rhabditophorans, trematodes

## Abstract

The second part of the catalogue of type material deposited in the Helminthological Collection of the Oswaldo Cruz Institute/ FIOCRUZ (CHIOC), between 1979 and 2016, is presented, to complement the first list of all types that was published in 1979. This part includes Platyhelminthes other than monogenoids, which were covered in the first part published in September 2016. The present catalogue comprises type material for 104 species, distributed across three classes, 40 families and 75 genera. Species names are listed systematically, followed by type host, infection site, type locality and specimens with their collection numbers and references. The classification and the nomenclature of the species have been updated.

## Introduction

The century-old Helminthological Collection of the Oswaldo Cruz Institute/ FIOCRUZ (CHIOC), Rio de Janeiro, Brazil, contains helminths that form part of the fauna of Brazil and other countries, from a wide range of hosts that were caught in different biomes. The samples are holotypes, paratypes and representative specimens of Platyhelminthes, Acanthocephala, Nematoda and other non-helminth phyla. Today, the CHIOC holds around 38.400 samples of helminth parasites from South America and other continents. It is the biggest collection in Latin America and it is among the largest collections at a worldwide reference level (Rego 1982, Knoff et al. 2010). Details about the history and composition of CHIOC were presented in Lopes et al. (2016).

The first catalogue of all type material held in the CHIOC recorded 719 types of helminths (only holotypes or type series): 408 of nematodes, 216 of digenetic trematodes, 11 of monogenoids, 52 of acanthocephalans, 28 of cestodes and four of pentastomids (Rego et al. 1979). Since publication of the first catalogue, the collection has grown substantially and the number of types has increased significantly as well. Recently, Lopes et al. (2016) published another catalogue listing 203 type species of Monogenoidea. The present catalogue is the second list of type species held in this collection, and it includes platyhelminths of the classes Rhabditophora, Trematoda and Cestoda that have been deposited in CHIOC since 1979. The purpose of this article is to inform the scientific community about the types deposited in CHIOC, up to 1 December 2016. In our presentation, we have followed the articles of the Code (ICZN 1999).

## Materials and methods

The specimens are stored in glass or plastic vials in 70% ethanol or as microscope slide preparations. All the material is available for consultation, but holotypes are not loaned. Unless otherwise stated, all type material is in good condition.

The catalogue is arranged taxonomically as classes, subclasses, orders, families, genera and species, under the original spelling and combinations. Classes and subclasses are arranged following phylogenetic order. Orders, families, genera and species are arranged alphabetically. The information on each entry is presented in the following format:

1. Original genus-species combination with author(s) and year of publication. Asterisk (*) denotes the type species of the genus.

2. Type host: updated scientific name, author(s) and year, with original scientific name in square brackets (when changed), followed by principal taxonomic group in parentheses.

3. Infestation or infection site in the host.

4. Type locality: country, province or state, department, specific locality and coordinates (if available).

5. Primary type status: CHIOC catalogue number. The categories for the types used followed articles 73–75 of the Code (ICZN 1999).

6. Remark sections are inserted when necessary and include additional information about host, locality or status of the species and types.

7. References include publications in which the species was described and those that mention type specimens in the CHIOC.

The valid names adopted for parasitized hosts follow specific bibliographies. Gastropod names are present in accordance with Rosenberg (2010); crustaceans, with Young (1998); caddisflies, with Paprocki and França (2014); stoneflies, with Froehlich (2010); bugs, with De Carlo (1967) and Ribeiro (2007); fish, with Froese and Pauly (2016); amphibians, with Frost (2006); reptiles, with Uetz et al. (2016); birds, with Lepage and Warnier (2014); and mammals, with Wilson and Reeder (2005). Mention of rhabditophorans, trematodes, cestodes and host species in this list does not imply that the authors of the present report agree with their validity or taxonomy. Some species catalogued have been synonymized and the comments about their taxonomy are in the remark sections. The higher classification of Rhabditophora follows Littlewood and Bray (2001) and Willems et al. (2006); Trematoda follows Gibson et al. (2002), Olson et al. (2003), Jones et al. (2005) and Bray et al. (2008); and Cestoda follows the Global Cestode Database (2016). The classification of families and genera follows specific references.

The following abbreviations are used in the text:

**BMNH** British Museum of Natural History, Collection at the Department of Zoology, Natural History Museum, London, England;

**CHFC** Helminthological Collection of the Science College, University of the Republic, Montevideo, Uruguay;

**CHIBB** Reference Helminthological Collection of the Parasitology Department, Bioscience Institute, Paulista State University (UNESP), Botucatu, São Paulo, Brazil;

**CHIOC** Helminthological Collection of the Oswaldo Cruz Institute, FIOCRUZ, Rio de Janeiro, Brazil;

**CHMLP** Helminthological Collection of the Museum of La Plata, La Plata, Buenos Aires, Argentina;

**CNHE** National Collection of Helminths, Institute of Biology, National Autonomous University of Mexico, Mexico City, Mexico;

**HWML** Harold W. Manter Laboratory, University of Nebraska State Museum, Lincoln, Nebraska, USA;

**INPA** National Institute for Amazon Research, Manaus, Amazonas, Brazil;

**IPCAS** Institute of Parasitology, Academy of Sciences of the Czech Republic, České Budějovice, Czech Republic;

**MEPN** Museum of the National Polytechnic School, Quito, Ecuador;

**MHNG** National Museum of Natural History, Geneva, Switzerland;

**MNHN** National Museum of Natural History, Paris, France;

**USNM** United States National Museum, Beltsville, Maryland, USA;

**USNPC** United States National Parasitological Collection, Beltsville, Maryland, USA.

## Results and discussion

This database and bibliographic survey presents the diversity of Rhabditophora, Trematoda (subclasses Aspidogastrea and Digenea) and Cestoda types in CHIOC, from Brazil and other countries in the world, covering more than 35 years of parasitological studies. We have now added 39 primary types of Rhabditophora, represented by eight species within one family (Temnocephalidae) and genus (*Temnocephala*); approximately 270 primary types of Trematoda, represented by 52 species distributed across 29 families and 46 genera; and approximately 130 primary types of Cestoda, represented by 44 species distributed across ten families and 28 genera. Among the trematodes, Rugogastridae was the only family of Aspidogastrea catalogued, while seven families of Digenea (Cryptogonimidae, Gorgoderidae, Haploporidae, Lecithasteridae, Lepocreadiidae, Monorchiidae and Opecoelidae) were the most representative, with three species each. Among the cestodes, Monticelliidae was the most representative family, with 21 species, followed by Proteocephalidae, with 13 species.

One hundred and four parasites of 70 species of vertebrate hosts and eight species of invertebrate hosts were catalogued. Most of the trematode and cestode type species recorded (84%) were parasites of bony fishes, as were the Monogenoidea (Lopes et al. 2016). Pimelodidae was the host family that presented the greatest diversity of parasites, with 23 species. Only five cestode species were parasites of cartilaginous fishes (Narcinidae, Potamotrygonidae, Triakidae and Urolophidae). The Tetrapoda hosts were frogs, birds, mammals and reptiles. One cestode species was a parasite of toads (Bufonidae) and one digenetic trematode species was a parasite of birds (Scolopacidae). Among the helminth parasites of mammals, one digenetic trematode species was a parasite of bats (Phyllostomidae), and one cestode and four digenetic trematodes species were parasites of rats (Cricetidae) and mice (Muridae). Among the helminth parasites of reptiles, five digenetic trematode species were parasites of alligators (Alligatoridae), two digenetic trematode species were parasites of lizards (Gekkonidae), two cestodes and one digenetic trematode species were parasites of snakes (Colubridae and Tropidophiidae), and one digenetic trematode species was a parasite of turtles (Podocnemididae). All temnocephalids were parasites of invertebrates: one species was a parasite of gastropods (Neritidae), one species was a parasite of caddisflies (Odontoceridae), one species was a parasite of stoneflies (Perlidae), two species were parasites of bugs (Belostomatidae and Naucoridae) and three species were parasites of freshwater crustaceans (Aeglidae and Trichodactylidae). Most of the species was collected in Brazil (90%), from all regions of this country except the northeast. Other samples were collected from Central America (Costa Rica) and South America (Bolivia, Ecuador, Paraguay and Uruguay).

### List of type species

#### Phylum Platyhelminthes Gegenbaur, 1859

##### Class Rhabditophora Ehlers, 1985 (‘Turbellaria’)

###### Order Rhabdocoela Ehrenberg, 1831

####### Temnocephalidae Monticelli, 1899

######## *Temnocephala* Blanchard, 1849

######### Temnocephala caddisflyi

Taxon classificationAnimaliaRhabdocoelaTemnocephalidae

Amato, Amato, Seixas, Vidigal & Andrade, 2011

########## Type host.

*Barypenthus* sp. (caddisfly larvae) (Trichoptera: Odontoceridae).

########## Infection site.

Adults on the dorsal and ventral sides of the thoracic segments; eggs predominantly on the dorsal side of the thoracic segments.

########## Type locality.

Brazil, Minas Gerais State, Jaboticatubas, Serra do Cipó National Park, State Highway MG–010 (km 96), Córrego das Pedras (19°22'10"S, 043°35'53"W).

########## Holotype.

CHIOC 37460 a.

########## Paratypes.

CHIOC 37460 b, 37461, 37462.

########## Remarks.

Other paratype deposited in CHMLP.

########## Reference.

Amato et al. (2011).

######### Temnocephala curvicirri

Taxon classificationAnimaliaRhabdocoelaTemnocephalidae

Amato & Amato, 2005

########## Type host.

*Belostoma dilatatum* (Dufour, 1863) (Hemiptera: Belostomatidae).

########## Infestation site.

Ventral side of body; eggs located under the rostrum and between the coxopodites of the first and second pairs of legs.

########## Type locality.

Brazil, Rio Grande do Sul State, Viamão, Tarumã Lake (30°04'14"S, 51°01'20"W).

########## Holotype.

CHIOC 36461.

########## Paratypes.

CHIOC 36462 a–g, 36463, 36464, 36465.

########## Remarks.

CHIOC 36463, 36464 and 36465 collected in Eldorado do Sul, Arrozeira (30°01'36"S, 51°22'42"W; Rio Grande do Sul State).

########## Reference.

Amato and Amato (2005).

######### Temnocephala cyanoglandula

Taxon classificationAnimaliaRhabdocoelaTemnocephalidae

Amato, Amato & Daudt, 2003

########## Type host.

*Aegla serrana* Buckup & Rossi, 1977 (Decapoda: Aeglidae).

########## Infection site.

Branchial chambers and body surface; eggs cemented over external surfaces of exoskeleton; in different regions of the ventral side: perioral area, pleural strips, esternal plates, pereiopods and chelipods; to a lesser extent on the dorsal side of cephalothorax and dorsal side of uropods.

########## Type locality.

Brazil, Rio Grande do Sul State, Cambará do Sul, Tainhas, 5 km East of State Road RS–020, Utopia II Farm (29°15'10"S, 50°15'45"W).

########## Holotype.

CHIOC 36199.

########## Paratypes.

CHIOC 36197, 36198, 36200, 36201, 36202.

########## Reference.

Amato et al. (2003).

######### Temnocephala euryhalina

Taxon classificationAnimaliaRhabdocoelaTemnocephalidae

Seixas, Amato & Amato, 2015

########## Type host.

*Neritina zebra* (Bruguière, 1792) (Gastropoda: Neritidae).

########## Infection site.

Adults and juveniles in mantle cavity; eggs not found in the body, on the shell, and neither on the operculum.

########## Type locality.

Brazil, Pará State, Cametá, Tocantins River (2°14'35.39"S, 49°29'50.29"W).

########## Holotype.

CHIOC 38046.

########## Remarks.

Paratypes deposited in CHMLP and INPA.

########## Reference.

Seixas et al. (2015a).

######### Temnocephala longivaginata

Taxon classificationAnimaliaRhabdocoelaTemnocephalidae

Seixas, Amato & Amato, 2011

########## Type host.

*Dilocarcinus septemdentatus* (Herbst, 1783) (Decapoda: Trichodactylidae).

########## Infection site.

Branchial chambers.

########## Type locality.

Brazil, Pará State, Peixe-Boi, Peixe-Boi River (01°07'17.65"S, 47°18'48.35"W).

########## Holotype.

CHIOC 37458 a.

########## Paratype.

CHIOC 37458 b.

########## Remarks.

Other paratype deposited in CHMLP.

########## Reference.

Seixas et al. (2011).

######### Temnocephala minutocirrus

Taxon classificationAnimaliaRhabdocoelaTemnocephalidae

Amato, Seixas & Amato, 2007

########## Type host.

*Cryphocricos granulosus* De Carlo, 1967 (Hemiptera: Naucoridae).

########## Infestation site.

Juvenile and adults on the ventral side of body. Eggs deposited on ventral side of body, between coxae, trochanters, and sternum; on dorsal side on top of scutellum, clavus, and hemelytra.

########## Type locality.

Brazil, Rio Grande do Sul State, Maquiné, Barra do Ouro, Arroio Forqueta (29°32'19"S, 50°14'47"W).

########## Holotype.

CHIOC 36877 a.

########## Paratypes.

CHIOC 36877 b, 36878, 36879, 36880, 36881.

########## Reference.

Amato et al. (2007).

######### Temnocephala trapeziformis

Taxon classificationAnimaliaRhabdocoelaTemnocephalidae

Amato, Amato & Seixas, 2006

########## Type host.

*Trichodactylus fluviatilis* Latreille, 1828 (Decapoda: Trichodactylidae).

########## Infestation site.

External surface of body, including the orbital cavities; eggs fixed over pleural sides of the carapace, orbital cavities, and mostly on the fourth pair of pereiopods.

########## Type locality.

Brazil, Rio Grande do Sul State, Maquiné, Arroio Água Parada (29°66'20"S, 50°21'15"W).

########## Holotype.

CHIOC 36617.

########## Paratypes.

CHIOC 36618, 36619, 36620, 36621, 36622, 36623.

########## Remarks.

Other paratypes deposited in the INPA collection.

########## Reference.

Amato et al. (2006).

######### Temnocephala stoneflyi

Taxon classificationAnimaliaRhabdocoelaTemnocephalidae

Seixas, Amato & Amato, 2015

########## Type host.

*Kempnyia reticulata* (Klapálek, 1916) (Plecoptera: Perlidae).

########## Infection site.

Head, mesonotum and median legs of the immatures.

########## Type locality.

Brazil, Espírito Santo State, Santa Teresa, Biological Reserve of Santa Lúcia, Córrego da Divisa (19°58'28.4"S; 40°31'54.4"W).

########## Holotype.

CHIOC 38203.

########## Paratype.

CHIOC 38204.

########## Reference.

Seixas et al. (2015b).

### Class Trematoda Rudolphi, 1808

#### Subclass Aspidogastrea Faust & Tang, 1936

##### Order Aspidogastrida Skrjabin & Guschanskaja, 1962

###### Rugogastridae Schell, 1973

####### *Rugogaster* Schell, 1973

######## Rugogaster callorhinchi

Taxon classificationAnimaliaAspidogastridaRugogastridae

Amato & Pereira Jr., 1995

######### Type host.

*Callorhinchus callorynchus* (Linnaeus, 1758) [= *Callorhinchus callorhynchus*] (Osteichthyes: Callorhinchidae).

######### Infection site.

Rectal glands.

######### Type locality.

Atlantic Ocean, coasts of Uruguay and Argentina, estuary of the La Plata River (34–36° S, 54–57° W).

######### Holotype.

CHIOC 33671 a.

######### Paratypes.

CHIOC 33671 b–s, 33672 a–c, 33673, 33674, 33675.

######### Reference.

Amato and Pereira Jr. (1995).

### Subclass Digenea Carus, 1863

#### Order Diplostomida Olson, Cribb, Tkach, Bray & Littlewood, 2003

##### Aporocotylidae Odhner, 1912

###### *Cardicola* Short, 1953

####### Cardicola brasiliensis

Taxon classificationAnimaliaDiplostomidaAporocotylidae

Knoff & Amato, 1992

######## Type host.

*Mugil liza* Valenciennes, 1836 [= *Mugil platanus* Günther, 1880] (Osteichthyes: Mugilidae).

######## Infection site.

Blood vessels of heart.

######## Type locality.

Brazil, Rio de Janeiro State, Sepetiba Bay (21°–23° S, 42°–45° W).

######## Holotype.

CHIOC 32657 a.

######## Paratype.

CHIOC 32657 b.

######## Remarks.

Other paratypes deposited in the HWML collection.

######## Reference.

Knoff and Amato (1992a).

### *Sanguinicola* Plehn, 1905

#### Sanguinicola platyrhynchi

Taxon classificationAnimaliaDiplostomidaAporocotylidae

Guidelli, Isaac & Pavanelli, 2002

##### Type host.

*Hemisorubim platyrhynchos* (Valenciennes, 1840) (Osteichthyes: Pimelodidae).

##### Infection site.

Visceral cavity.

##### Type locality.

Brazil, Mato Grosso do Sul State, floodplain of the upper Paraná River, Baía River.

##### Holotype.

CHIOC 34360.

##### Paratypes.

CHIOC 34361 a–b.

##### Reference.

Guidelli et al. (2002).

### Proterodiplostomidae Dubois, 1936

#### *Proterodisplostomum* Dubois, 1936

##### Proterodisplostomum breve

Taxon classificationAnimaliaDiplostomidaProterodiplostomidae

Catto & Amato, 1994

###### Type host.

*Caiman yacare* (Daudin, 1802) [= *Caiman crocodilus yacare*] (Crocodylia: Alligatoridae).

###### Infection site.

Anterior and middle sections of the small intestine.

###### Type locality.

Brazil, Mato Grosso do Sul State, Corumbá, Nhumirim Farm (18°59'S, 56°39'W).

###### Holotype.

CHIOC 33001 c.

###### Paratypes.

CHIOC 33001 a–b, 33002 a–b, 33003 a–d, 33004.

###### Remarks.

Other paratypes deposited in the USNM collection.

###### Reference.

Catto and Amato (1994).

##### Proterodisplostomum globulare

Taxon classificationAnimaliaDiplostomidaProterodiplostomidae

Catto & Amato, 1994

###### Type host.

*Caiman yacare* (= *C. crocodilus yacare*)

###### Infection site.

Anterior, middle, and posterior sections of the small intestine.

###### Type locality.

Brazil, Mato Grosso do Sul State, Corumbá, Nhumirim Farm (18°59'S, 56°39'W).

###### Holotype.

CHIOC 33005 b.

###### Paratypes.

CHIOC 33005 a, 33006, 33007, 33008 a–b, 33009, 33010 a–b.

###### Remarks.

Other paratypes deposited in the USNM collection.

###### Reference.

Catto and Amato (1994).

### Order Plagiorchiida La Rue, 1957

#### Acanthocolpidae Lühe, 1906

##### *Tormopsolus* Poche, 1926

###### Tormopsolus brasiliensis

Taxon classificationAnimaliaPlagiorchiidaAcanthocolpidae

Knoff & Fernandes, 2010

####### Type host.

*Genypterus brasiliensis* Regan, 1903 (Osteichthyes: Ophidiidae).

####### Infection site.

Intestine.

####### Type locality.

Brazil, Rio de Janeiro State, Niterói (22°53'00"S, 43°06'16"W).

####### Holotype.

CHIOC 37228 a.

####### Paratypes.

CHIOC 37228 b–l, 37229, 37230, 37231 a–b.

####### Reference.

Knoff and Fernandes (2010).

### Alloglossidiidae Hernández-Mena, Mendoza-Garfias, Ornelas-García & Ponce de León, 2016

#### *Magnivitellinum* Kloss, 1966

##### Magnivitellinum corvitellinum

Taxon classificationAnimaliaPlagiorchiidaAlloglossidiidae

Lacerda, Takemoto & Pavanelli, 2009

###### Type host.

*Hoplosternum littorale* (Hancock, 1828) (Osteichthyes: Callichthyidae).

###### Infection site.

Intestine.

###### Type locality.

Brazil, Paraná State, Upper Paraná River floodplain (22°43'S, 53°10'W).

###### Holotype.

CHIOC 37170 a.

###### Paratype.

CHIOC 37170 b.

###### Remarks.

Other paratype deposited in USNPC.

###### Reference.

Lacerda et al. (2009).

### Anenterotrematidae Yamaguti, 1958

#### *Apharyngotrema* Marshall & Miller, 1979

##### Apharyngotrema lenti

Taxon classificationAnimaliaPlagiorchiidaAnenterotrematidae

Santos & Gibson, 1998

###### Type host.

*Anoura caudifer* (Geoffroy, 1818) (Chiroptera: Phyllostomidae).

###### Infection site.

Gall-bladder.

###### Type locality.

Brazil, Amapá State, Vila Nova, Serra do Navio (0°37'N, 51°47'W).

###### Holotype.

CHIOC 33765.

###### Paratypes.

CHIOC 33766 a–b.

###### Remarks.

CHIOC 33766 a–b collected from *Saccopteryx bilineata* (Temminck, 1838) (Chiroptera: Emballonuridae). Other paratypes deposited in the BMNH collection.

###### Reference.

Santos and Gibson (1998).

### Apocreadiidae Skrjabin, 1942

#### *Homalometron* Stafford, 1904

##### Homalometron carapevae

Taxon classificationAnimaliaPlagiorchiidaApocreadiidae

Amato, 1983

###### Type host.

*Eugerres brasilianus* (Cuvier, 1830) (Osteichthyes: Gerreidae).

###### Infection site.

Intestine.

###### Type locality.

Brazil, Santa Catarina State, Florianópolis, Lagoa da Conceição.

###### Holotype.

CHIOC 31932.

###### Remarks.

Other material deposited in the HWML collection.

###### Reference.

Amato (1983a).

### *Neoapocreadium* Siddiqi & Cable, 1960

#### Neoapocreadium chabaudi

Taxon classificationAnimaliaPlagiorchiidaApocreadiidae

Kohn & Fernandes, 1982

##### Type host.

*Stephanolepis hispidus* (Linnaeus, 1766) (Osteichthyes: Monacanthidae).

##### Infection site.

Intestine.

##### Type locality.

Brazil, Rio de Janeiro State, Angra do Reis.

##### Holotype.

CHIOC 31813 b.

##### Paratype.

CHIOC 31813 a.

##### Reference.

Kohn and Fernandes (1982a).

### Bucephalidae Poche, 1907

#### *Rhipidocotyle* Diesing, 1858

##### Rhipidocotyle gibsoni

Taxon classificationAnimaliaPlagiorchiidaBucephalidae

Kohn & Fernandes, 1994

###### Type host.

*Acestrorhynchus lacustris* (Lütken, 1875) (Osteichthyes: Acestrorhynchidae).

###### Infection site.

Pyloric caeca.

###### Type locality.

Brazil, Paraná State, Paraná River, Guaíra.

###### Holotype.

CHIOC 33119 a.

###### Paratypes.

CHIOC 33119 b–m, 33120 a–b.

###### Remarks.

Other paratypes deposited in the BMNH collection.

###### Reference.

Kohn and Fernandes (1994).

### Cladorchiidae Fischoeder, 1901

#### *Dadayius* Fukui, 1929

##### Dadayius pacupeva

Taxon classificationAnimaliaPlagiorchiidaCladorchiidae

Lacerda, Takemoto & Pavanelli, 2003

###### Type host.

*Metynnis maculatus* (Kner, 1858) (Osteichthyes: Serrasalmidae).

###### Infection site.

Intestinal tract.

###### Type locality.

Brazil, Paraná State, Upper Paraná River floodplain near Porto Rico.

###### Holotype.

CHIOC 36394 a.

###### Paratypes.

CHIOC 36394 b, 36395.

###### Reference.

Lacerda et al. (2003).

### *Oriximinatrema* Knoff, Brooks, Mullins & Gomes, 2012

#### * Oriximinatrema noronhae

Taxon classificationAnimaliaPlagiorchiidaCladorchiidae

Knoff, Brooks, Mullins & Gomes, 2012

##### Type host.

*Podocnemis expansa* (Schweigger, 1812) (Testudines: Podocnemididae).

##### Infection site.

Stomach and intestine.

##### Type locality.

Brazil, Pará State, Oriximiná (01°45'55"S, 55°51'50"W).

##### Holotype.

CHIOC 36633 a.

##### Paratypes.

CHIOC 36633 b, 36634 a–g, 36635.

##### Remarks.

There is no paratype CHIOC 36634 “h” as informed in the original description.

##### Reference.

Knoff et al. (2012).

### Cryptogonimidae Ward, 1917

#### *Annakohniella* Fernandes, Cohen, Mendonça & Justo, 2013

##### * Annakohniella travassosi

Taxon classificationAnimaliaPlagiorchiidaCryptogonimidae

Fernandes, Cohen, Mendonça & Justo, 2013

###### Type host.

*Rhaphiodon vulpinus* Agassiz, 1829 (Osteichthyes: Cynodontidae).

###### Infection site.

Intestine.

###### Type locality.

Brazil, Tocantins State, Capivara River (81°27'57"S, 31°44'23"W).

###### Holotype.

CHIOC 37532 a.

###### Paratypes.

CHIOC 37532 b–g.

###### Reference.

Fernandes et al. (2013a).

### *Parspina* Pearse, 1920

#### Parspina papernai

Taxon classificationAnimaliaPlagiorchiidaCryptogonimidae

Kohn & Fernandes, 2011

##### Type host.

*Iheringichthys labrosus* (Lütken, 1874) (Osteichthyes: Pimelodidae).

##### Infection site.

Intestine.

##### Type locality.

Brazil, Paraná State, reservoir of the Itaipu Binational Hydroelectric Power Station, Guaíra (24°04'48"S, 54°15'21"W).

##### Holotype.

CHIOC 37313 a.

##### Paratypes.

CHIOC 37312 a–c, 37313 b–c.

##### Reference.

Kohn and Fernandes (2011).

### *Proctocaecum* Baugh, 1957

#### Proctocaecum dorsale

Taxon classificationAnimaliaPlagiorchiidaCryptogonimidae

Catto & Amato, 1993

##### Type host.

*Caiman yacare* (= *C. crocodilus yacare*)

##### Infection site.

Intestine.

##### Type locality.

Brazil, Mato Grosso do Sul State, Corumbá, Nhumirim Farm (18°59'S, 56°39'W).

##### Holotype.

CHIOC 32896 a.

##### Paratypes.

CHIOC 32896 b–c, 32897 a–b, 32898, 32899 a–b, 33000 a–b.

##### Remarks.

Other paratypes deposited in the USNM collection.

##### Reference.

Catto and Amato (1993a).

### Dicrocoeliidae Looss, 1899

#### *Yungasicola* Gardner & Pérez-Léon, 2002

##### * Yungasicola travassosi

Taxon classificationAnimaliaPlagiorchiidaDicrocoeliidae

Gardner & Pérez-Léon, 2002

###### Type host.

*Akodon fumeus* Thomas, 1902 (Rodentia: Cricetidae).

###### Infection site.

Gall bladder and bile ducts.

###### Type locality.

Bolivia, La Paz, Aceromarca River (16°19'S, 67°53'W), elevation 2.990 m.

###### Paratype.

CHIOC 36829.

###### Remarks.

Holotype and paratypes deposited in the HWML collection. Other paratypes deposited in CNHE and USNPC. Paratype from CHIOC donated by Gardner.

###### Reference.

Gardner and Pérez-Léon (2002).

### Didymozoidae Monticelli, 1888

#### *Didymocystis* Ariola, 1902

##### Didymocystis lamotheargumedoi

Taxon classificationAnimaliaPlagiorchiidaDidymozoidae

Kohn & Justo, 2008

###### Type host.

*Thunnus atlanticus* (Lesson, 1831) (Osteichthyes: Scombridae).

###### Infection site.

Operculum and palate.

###### Type locality.

Brazil, Rio de Janeiro State, off Cabo Frio (22°52'46"S, 42°01'07"W).

###### Holotype.

CHIOC 36927 a.

###### Paratypes.

CHIOC 36927 b–p, 36928 a–b, 39929 a–b, 36930, 36931, 36932 a–d, 36933.

###### Remarks.

Paratypes collected from other scombrid fish: CHIOC 36928 a–b from the operculum of *Katsuwonus pelamis* (Linnaeus, 1758), and 36933 from the palate of *Thunnus albacares* (Bonnaterre, 1788).

###### Reference.

Kohn and Justo (2008).

### *Pozdnyakovia* Justo & Kohn, 2012

#### * Pozdnyakovia gibsoni

Taxon classificationAnimaliaPlagiorchiidaDidymozoidae

Justo & Kohn, 2012

##### Type host.

*Katsuwonus pelamis*

##### Infection site.

Serosa of stomach.

##### Type locality.

Brazil, Rio de Janeiro State, off Cabo Frio (22°52'46"S, 42°01'07"W).

##### Holotype.

CHIOC 37541.

##### Paratypes.

CHIOC 37542 a–c.

##### Reference.

Justo and Kohn (2012).

### Echinostomatidae Looss, 1899

#### *Echinostoma* Rudolphi, 1809

##### Echinostoma luisreyi

Taxon classificationAnimaliaPlagiorchiidaEchinostomatidae

Maldonado Jr., Vieira & Lanfredi, 2003

###### Type host.

*Mus musculus* Linnaeus, 1758 (Rodentia: Muridae).

###### Infection site.

Posterior part of the small intestine.

###### Type locality.

Brazil, Rio de Janeiro State, Sumidouro (22°02"S, 42°41"W).

###### Holotype.

CHIOC 34214 a.

###### Paratypes.

CHIOC 34214 b–d.

###### Remarks.

Other paratype deposited in USNPC.

###### Reference.

Maldonado Jr. et al. (2003).

### Fellodistomidae Nicoll, 1909

#### *Monascus* Looss, 1907

##### Monascus americanus

Taxon classificationAnimaliaPlagiorchiidaFellodistomidae

Amato, 1982

###### Type host.

*Trachurus lathami* Nichols, 1920 (Osteichthyes: Carangidae).

###### Infection site.

Intestine.

###### Type locality.

Brazil, Santa Catarina State, Florianópolis, Barra da Lagoa.

###### Holotype.

CHIOC 31869.

###### Remarks.

Paratype deposited in the HWML collection. This species was considered to be a synonym of *M. filiformis* (Rudolphi, 1819) by Gaevskaya (1989) and Sey et al. (2003). However, the species is accepted by Gibson (2015).

###### References.

Amato (1982a), Gaevskaya (1989), Sey et al. (2003), Gibson (2015).

### *Tergestia* Stossich, 1899

#### Tergestia selenei

Taxon classificationAnimaliaPlagiorchiidaFellodistomidae

Amato, 1982

##### Type host.

*Selene vomer* (Linnaeus, 1758) (Osteichthyes: Carangidae).

##### Infection site.

Intestine and pyloric ceca.

##### Type locality.

Brazil, Santa Catarina State, Florianópolis, Barra da Lagoa.

##### Holotype.

CHIOC 31867.

##### Remarks.

Paratype deposited in the HWML collection. The species was considered to be a synonym of *T. pauca* Freitas & Kohn, 1965 by Wallet and Kohn (1987). However, the species is accepted by Gibson (2015).

##### References.

Amato (1982a), Wallet and Kohn (1987), Gibson (2015).

### Gorgoderidae Looss, 1899

#### *Dendrorchis* Travassos, 1926

##### Dendrorchis retrobiloba

Taxon classificationAnimaliaPlagiorchiidaGorgoderidae

Volonterio & León, 2005

###### Type host.

*Astyanax fasciatus* (Cuvier, 1819) (Osteichthyes: Characidae).

###### Infection site.

Swim bladder.

###### Type locality.

Uruguay, Montevideo, Cañada del Dragón stream (34°47'S, 56°14'W)

###### Paratypes.

CHIOC 36392 a–b.

###### Remarks.

Holotype and paratypes deposited in CHFC. Other paratypes deposited in CNHE.

###### Reference.

Volonterio and León (2005).

### *Phyllodistomum* Braun, 1899

#### Phyllodistomum mugilis

Taxon classificationAnimaliaPlagiorchiidaGorgoderidae

Knoff & Amato, 1992

##### Type host.

*Mugil liza* (= *M. platanus*)

##### Infection site.

Urinary bladder.

##### Type locality.

Brazil, Rio de Janeiro State, Guanabara Bay.

##### Holotype.

CHIOC 32658.

##### Remarks.

Paratype deposited in the HWML collection.

##### Reference.

Knoff and Amato (1992b).

#### Phyllodistomum rhamdiae

Taxon classificationAnimaliaPlagiorchiidaGorgoderidae

Amato & Amato, 1993

##### Type host.

*Rhamdia quelen* (Quoy & Gaimard, 1824) (Osteichthyes: Heptapteridae).

##### Infection site.

Urinary bladder.

##### Type locality.

Brazil, Rio de Janeiro State, Guandu River, Km 47 old Rio de Janeiro-São Paulo highway.

##### Holotype.

CHIOC 33067.

##### Paratype.

CHIOC 33068.

##### Remarks.

Other paratypes deposited in the USNM collection.

##### Reference.

Amato and Amato (1993).

### Haploporidae Nicoll, 1914

#### *Chalcinotrema* Freitas, 1947

##### Chalcinotrema thatcheri

Taxon classificationAnimaliaPlagiorchiidaHaploporidae

Kohn, Fernandes & Gibson, 1999

###### Type host.

*Schizodon knerii* (Steindachner, 1875) (Osteichthyes: Anostomidae).

###### Infection site.

Intestine.

###### Type locality.

Brazil, Paraná State, Paraná River, Guaíra.

###### Holotype.

CHIOC 33920 a.

###### Paratypes.

CHIOC 33920 b–d, 33921.

###### Reference.

Kohn et al. (1999).

### *Intromugil* Overstreet & Curran, 2005

#### Intromugil annakohnae

Taxon classificationAnimaliaPlagiorchiidaHaploporidae

Fernandes & Cohen, 2006

##### Type host.

*Mugil liza*

##### Infection site.

Intestine.

##### Type locality.

Brazil, Rio de Janeiro State, Rio de Janeiro, Copacabana beach (22°58'0"S, 43°10'0"W).

##### Holotype.

CHIOC 36639 a.

##### Paratype.

CHIOC 36639 b.

##### Reference.

Fernandes and Cohen (2006).

### *Saccocoelioides* Szidat, 1954

#### Saccocoelioides godoyi

Taxon classificationAnimaliaPlagiorchiidaHaploporidae

Kohn & Fróes, 1986

##### Type host.

*Leporinus elongatus* Valenciennes, 1850 (Osteichthyes: Anostomidae).

##### Infection site.

Intestine and stomach.

##### Type locality.

Brazil, Rio Grande do Sul State, Guaíba estuary.

##### Holotype.

CHIOC 32185 a.

##### Paratypes.

CHIOC 32185 b–h.

##### Reference.

Kohn and Fróes (1986).

### Haplosplanchnidae Poche, 1926

#### *Schikhobalotrema* Skrjabin & Guschanskaja, 1955

##### Schikhobalotrema solitaria

Taxon classificationAnimaliaPlagiorchiidaHaplosplanchnidae

Fernandes & Goulart, 1989

###### Type host.

*Stephanolepsis hispidus* (Linnaeus, 1766) (Osteichthyes: Monacanthidae).

###### Infection site.

Intestine.

###### Type locality.

Brazil, Rio de Janeiro State, Rio de Janeiro, Copacabana beach.

###### Holotype.

CHIOC 32583.

###### Reference.

Fernandes and Goulart (1989a).

### Hemiuridae Looss, 1899

#### *Dinosoma* Manter, 1934

##### Dinosoma clupeola

Taxon classificationAnimaliaPlagiorchiidaHemiuridae

Fernandes & Goulart, 1989

###### Type host.

*Harengula clupeola* (Cuvier, 1829) (Osteichthyes: Clupeidae).

###### Infection site.

Stomach and intestine.

###### Type locality.

Brazil, Rio de Janeiro State, Rio de Janeiro, Copacabana beach.

###### Holotype.

CHIOC 32479.

###### Paratypes.

CHIOC 32480 a–b, 32481 a–b, 32482, 32483.

###### Reference.

Fernandes and Goulart (1989b).

### *Lecithocladium* Lühe, 1901

#### Lecithocladium chaetodipteri

Taxon classificationAnimaliaPlagiorchiidaHemiuridae

Amato, 1983

##### Type host.

*Chaetodipterus faber* (Broussonet, 1782) (Osteichthyes: Ephippidae).

##### Infection site.

Stomach.

##### Type locality.

Brazil, Santa Catarina State, Florianópolis, Barra da Lagoa.

##### Holotype.

CHIOC 31992.

##### Remarks.

Other material deposited in the HWML collection.

##### Reference.

Amato (1983b).

### Lecithasteridae Odhner, 1905

#### *Hysterolecitha* Linton, 1910

##### Hysterolecitha brasiliensis

Taxon classificationAnimaliaPlagiorchiidaLecithasteridae

Oliveira, Amato & Knoff, 1988

###### Type host.

*Mugil liza*

###### Infection site.

Stomach and intestine.

###### Type locality.

Brazil, Rio de Janeiro State, Itaguaí, Da Guarda River (22°45’–23°00'S, 43°50'W).

###### Holotype.

CHIOC 32287.

###### Paratypes.

CHIOC 32288 a–b.

###### Remarks.

Other paratype deposited in the HWML collection.

###### Reference.

Oliveira et al. (1988).

### *Lecithaster* Lühe, 1901

#### Lecithaster falcatus

Taxon classificationAnimaliaPlagiorchiidaLecithasteridae

Amato, 1983

##### Type host.

*Diplectrum radiale* (Quoy & Gaimard, 1824) (Osteichthyes: Serranidae).

##### Infection site.

Stomach and intestine.

##### Type locality.

Brazil, Santa Catarina State, Florianópolis, Norte Bay, Sambaqui.

##### Holotype.

CHIOC 31988.

##### Remarks.

Paratype deposited in the HWML collection.

##### Reference.

Amato (1983b).

### *Prolecitha* Manter, 1961

#### Prolecitha brasiliensis

Taxon classificationAnimaliaPlagiorchiidaLecithasteridae

Amato, 1983

##### Type host.

*Trachinotus marginatus* Cuvier, 1832 (Osteichthyes: Carangidae).

##### Infection site.

Intestine.

##### Type locality.

Brazil, Santa Catarina State, Florianópolis, Barra da Lagoa.

##### Holotype.

CHIOC 31991.

##### Remarks.

No paratype deposited.

##### Reference.

Amato (1983b).

### Lepocreadiidae Odhner, 1905

#### *Multitestoides* Yamaguti, 1971

##### Multitestoides brasiliensis

Taxon classificationAnimaliaPlagiorchiidaLepocreadiidae

(Amato, 1983)

###### Type host.

*Chaetodipterus faber*

###### Infection site.

Intestine.

###### Type locality.

Brazil, Santa Catarina State, Florianópolis, Norte Bay, Sambaqui.

###### Holotype.

CHIOC 31938.

###### Paratype.

CHIOC 31939.

###### Remarks.

Other paratypes deposited in the HWML collection. Species described as Multitestis (Multitestoides) brasiliensis by Amato (1983a).

###### Reference.

Amato (1983a).

### *Prodistomum* Linton, 1910

#### Prodistomum chloroscombri

Taxon classificationAnimaliaPlagiorchiidaLepocreadiidae

(Amato, 1983)

##### Type host.

*Chloroscombrus chrysurus* (Linnaeus, 1766) (Osteichthyes: Carangidae).

##### Infection site.

Intestine and pyloric ceca.

##### Type locality.

Brazil, Santa Catarina State, Florianópolis, Norte Bay, Sambaqui.

##### Holotype.

CHIOC 31933.

##### Remarks.

Paratypes deposited in the HWML collection. Species described as *Acanthocolpoides chloroscombri* by Amato (1983a).

##### Reference.

Amato (1983a).

#### Prodistomum pomatomi

Taxon classificationAnimaliaPlagiorchiidaLepocreadiidae

(Amato, 1983)

##### Type host.

*Pomatomus saltatrix* (Linnaeus, 1766) (Osteichthyes: Pomatomidae).

##### Infection site.

Intestine.

##### Type locality.

Brazil, Santa Catarina State, Florianópolis, Norte Bay, Biguaçú.

##### Holotype.

CHIOC 31934.

##### Remarks.

Paratype deposited in the HWML collection. Species described as *Acanthocolpoides pomatomi* by Amato (1983a).

##### Reference.

Amato (1983a).

### Microphallidae Ward, 1901

#### *Metamaritrema* Yamaguti, 1971

##### Metamaritrema mariettavogeae

Taxon classificationAnimaliaPlagiorchiidaMicrophallidae

(Kohn, Fernandes, Pinto & Mello, 1981)

###### Type host.

*Nectomys squamipes* (Brants, 1827) (Rodentia: Cricetidae).

###### Infection site.

Intestine.

###### Type locality.

Brazil, Goiás State, Formosa.

###### Holotype.

CHIOC 31877 a.

###### Paratypes.

CHIOC 37877 b–q.

###### Remarks.

Species referred to as *Maritremopsis mariettavogeae* by Kohn et al. (1981). *Maritremopsis* was considered to be a synonym of *Metamaritrema* by Deblock (2008).

###### References.

Kohn et al. (1981), Deblock (2008).

### Monorchiidae Odner, 1911

#### *Diplomonorchis* Hopkins, 1941

##### Diplomonorchis catarinensis

Taxon classificationAnimaliaPlagiorchiidaMonorchiidae

Amato, 1982

###### Type host.

*Micropogonias furnieri* (Desmarest, 1823) (Osteichthyes: Sciaenidae).

###### Infection site.

Pyloric ceca and intestine.

###### Type locality.

Brazil, Santa Catarina State, Florianópolis, Barra Norte.

###### Holotype.

CHIOC 31910.

###### Remarks.

Paratype deposited in the HWML collection.

###### Reference.

Amato (1982b).

### *Genolopa* Linton, 1910

#### Genolopa mugilis

Taxon classificationAnimaliaPlagiorchiidaMonorchiidae

Knoff & Amato, 1992

##### Type host.

*Mugil liza* (= *M. platanus*)

##### Infection site.

Stomach and intestine.

##### Type locality.

Brazil, Rio de Janeiro State, Sepetiba Bay, Itacuruçá.

##### Holotype.

CHIOC 32659.

##### Paratype.

CHIOC 32660.

##### Remarks.

Paratype from CHIOC collected in Itaipú, Niterói (Rio de Janeiro State). Other paratypes deposited in the HWML collection.

##### Reference.

Knoff and Amato (1992c).

### *Parahurleytrema* Nahhas & Powell, 1965

#### Parahurleytrema catarinense

Taxon classificationAnimaliaPlagiorchiidaMonorchiidae

(Amato, 1982) Nahhas & Grewal, 1997

##### Type host.

*Trachinotus carolinus* (Linnaeus, 1766)

##### Infection site.

Intestine.

##### Type locality.

Brazil, Santa Catarina State, Florianópolis, Barra da Lagoa.

##### Holotype.

CHIOC 31874.

##### Remarks.

Paratype deposited in the HWML collection. Species described as *Hurleytrema catarinensis* by Amato (1982b).

##### References.

Amato (1982b), Nahhas and Grewal (1997).

### Opecoelidae Ozaki, 1925

#### *Opecoeloides* Odhner, 1928

##### Opecoeloides catarinensis

Taxon classificationAnimaliaPlagiorchiidaOpecoelidae

Amato, 1983

###### Type host.

*Micropogonias furnieri*

###### Infection site.

Intestine.

###### Type locality.

Brazil, Santa Catarina State, Florianópolis, Norte Bay, Sambaqui.

###### Holotype.

CHIOC 31941 a.

###### Paratype.

CHIOC 31941 b.

###### Remarks.

Other paratype deposited in the HWML collection.

###### Reference.

Amato (1983a).

##### Opecoeloides melanopteri

Taxon classificationAnimaliaPlagiorchiidaOpecoelidae

Amato, 1983

###### Type host.

*Eucinostomus melanopterus* (Bleeker, 1863) (Osteichthyes: Gerreidae).

###### Infection site.

Intestine.

###### Type locality.

Brazil, Santa Catarina State, Florianópolis, Norte Bay, Sambaqui.

###### Holotype.

CHIOC 31942.

###### Remarks.

Paratype deposited in the HWML collection.

###### Reference.

Amato (1983a).

##### Opecoeloides stenosomae

Taxon classificationAnimaliaPlagiorchiidaOpecoelidae

Amato, 1983

###### Type host.

*Micropogonias furnieri*

###### Infection site.

Intestine.

###### Type locality.

Brazil, Santa Catarina State, Florianópolis, Norte Bay, Sambaqui.

###### Holotype.

CHIOC 31943.

###### Remarks.

Paratype deposited in the HWML collection.

###### Reference.

Amato (1983a).

### Opisthorchiidae Looss, 1899

#### *Amphimerus* Barker, 1911

##### Amphimerus bragai

Taxon classificationAnimaliaPlagiorchiidaOpisthorchiidae

Moraes Neto, Thatcher & Lanfredi, 1998

###### Type host.

*Nectomys squamipes*

###### Infection site.

Bile ducts.

###### Type locality.

Brazil, Minas Gerais State, Lagoa Santa (19°38'S, 43°53'W).

###### Holotype and paratype.

CHIOC 33645 a–b.

###### Remarks.

Holotype and paratype in the same vial.

###### Reference.

Moraes Neto et al. (1998).

### Philophthalmidae Looss, 1899

#### *Oswaldotrema* Muniz-Pereira & Pinto, 2000

##### * Oswaldotrema nacinovici

Taxon classificationAnimaliaPlagiorchiidaPhilophthalmidae

Muniz-Pereira & Pinto, 2000

###### Type host.

*Numenius phaeopus* (Linnaeus, 1758) (Aves: Scolopacidae).

###### Infection site.

Anterior intestine.

###### Type locality.

Brazil, Rio de Janeiro State, Rio de Janeiro, Pedra de Guaratiba, mouth of Piraque River.

###### Holotype.

CHIOC 34195.

###### Remarks.

No paratype deposited.

###### Reference.

Muniz-Pereira and Pinto (2000).

### Plagiorchiidae Lühe, 1901

#### *Haplometroides* Odner, 1910

##### Haplometroides intercaecalis

Taxon classificationAnimaliaPlagiorchiidaPlagiorchiidae

Silva, Ferreira & Strüssmann, 2007

###### Type host.

*Phalotris nasutus* (Gomes, 1915) (Serpentes: Colubridae).

###### Infection site.

Esophagus, stomach, and small intestine.

###### Type locality.

Brazil, Mato Grosso do Sul State, Corumbá, Nhecolândia region, Nhumirim Farm (18°59'0"S, 56°39'0"W).

###### Holotype.

CHIOC 36810.

###### Remarks.

Paratypes deposited in CHIBB.

###### Reference.

Silva et al. (2007).

### *Plagiorchis* Lühe, 1899

#### Plagiorchis vicentei

Taxon classificationAnimaliaPlagiorchiidaPlagiorchiidae

Rodrigues, 1994

##### Type host.

*Hemidactylus mabouia* (Moreau de Jonnès, 1818) (Gekkota: Gekkonidae).

##### Infection site.

Small intestine.

##### Type locality.

Brazil, Rio de Janeiro State, Teresópolis.

##### Holotype.

CHIOC 32847 c.

##### Paratypes.

CHIOC 32847 a–b, d.

##### Reference.

Rodrigues (1994).

### Styphlotrematidae Baer, 1924

#### *Styphlotrema* Odhner, 1911

##### Styphlotrema artigasi

Taxon classificationAnimaliaPlagiorchiidaStyphlotrematidae

Kohn & Fernandes, 1982

###### Type host.

*Guavina guavina* (Valenciennes, 1837) (Osteichthyes: Eleotridae).

###### Infection site.

Intestine.

###### Type locality.

Brazil, Rio de Janeiro State, Guanabara Bay.

###### Holotype.

CHIOC 31819.

###### Reference.

Kohn and Fernandes (1982b).

### Telorchiidae Looss, 1899

#### *Pseudotelorchis* Yamaguti, 1971

##### Pseudotelorchis caimanis

Taxon classificationAnimaliaPlagiorchiidaTelorchiidae

Catto & Amato, 1993

###### Type host.

*Caiman yacare* (= *C. crocodilus yacare*)

###### Infection site.

Oviduct, near the opening of the cloaca.

###### Type locality.

Brazil, Mato Grosso do Sul State, Corumbá, Nhumirim Farm (18°06'S, 56°36'W).

###### Holotype.

CHIOC 32889 a.

###### Paratypes.

CHIOC 32889 b–c.

###### Remarks.

Other paratype deposited in the USNM collection.

###### Reference.

Catto and Amato (1993a).

##### Pseudotelorchis yacarei

Taxon classificationAnimaliaPlagiorchiidaTelorchiidae

Catto & Amato, 1993

###### Type host.

*Caiman yacare* (= *C. crocodilus yacare*)

###### Infection site.

Intestine.

###### Type locality.

Brazil, Mato Grosso do Sul State, Corumbá, Nhumirim Farm (18°06'S, 56°36'W).

###### Holotype.

CHIOC 32890 a.

###### Paratypes.

CHIOC 32890 b–c, 32891, 32892 a–c, 32893, 32894.

###### Remarks.

CHIOC 32892 a–c and 32894 a–b were collected in Santana Farm (18° 06'S, 56°36'W), Corumbá. Other paratype deposited in the USNM collection.

###### Reference.

Catto and Amato (1993b).

### Zoogonidae Odhner, 1902

#### *Porangatus* Fernandes, Malta & Morais, 2013

##### * Porangatus ceteyus

Taxon classificationAnimaliaPlagiorchiidaZoogonidae

Fernandes, Malta & Morais, 2013

###### Type host.

*Hoplosternum littorale*

###### Infection site.

Intestine.

###### Type locality.

Brazil, Amazonas State, Catalão Lake (03°09'47"S, 59°54'29"W).

###### Holotype.

CHIOC 37543 a.

###### Paratypes.

CHIOC 37543 b–v.

###### Reference.

Fernandes et al. (2013b).

### Class Cestoda Rudophi, 1819

#### Order Cyclophyllidea Braun, 1900

##### Davaineidae Braun, 1900

###### Raillietina (Raillietina) Fuhrmann, 1920

####### Raillietina (Raillietina) guaricanae

Taxon classificationAnimaliaCyclophyllideaDavaineidae

César & Luz, 1993

######## Type host.

*Oryzomys ratticeps* (Hensel, 1873) (Rodentia: Cricetidae).

######## Infection site.

Small intestine.

######## Type locality.

Brazil, Paraná State, Enviromnetal Protection Area of Guaricana (25°40'S, 48°55'W).

######## Holotype.

CHIOC 33043 a–c.

######## Paratypes.

CHIOC 33043 d–f, 33044.

######## Reference.

César and Luz (1993).

### Nematotaeniidae Lühe, 1910

#### *Lanfrediella* Melo, Giese, Furtado, Soares, Gonçalves, Vallinoto & Santos, 2011

##### * Lanfrediella amphicirrus

Taxon classificationAnimaliaCyclophyllideaNematotaeniidae

Melo, Giese, Furtado, Soares, Gonçalves, Vallinoto & Santos, 2011

###### Type host.

*Rhinella marina* (Linnaeus, 1758) (Amphibia: Bufonidae).

###### Infection site.

Small intestine.

###### Type locality.

Brazil, Pará State, Belém (01°28'03"S, 48°20'18"W).

###### Holotype.

CHIOC 37314 a.

###### Paratypes.

CHIOC 35706, 37314 b.

###### Reference.

Melo et al. (2011).

### Order Onchoproteocephalidea Caira, Jensen, Waeschenbach, Olson & Littlewood, 2014

#### Oncobothriidae Braun, 1900

##### *Acanthobothrium* van Beneden, 1849

###### Acanthobothrium inbiorum

Taxon classificationAnimaliaOnchoproteocephalideaOncobothriidae

Marques, Centritto & Stewart, 1997

####### Type host.

*Narcine entemedor* Jordan & Starks, 1895 (Chondrichthyes: Narcinidae).

####### Infection site.

Spiral valve.

####### Type locality.

Costa Rica, Guanacaste, Gulf of Santa Helena, Cuajiniquil beach (10°57'N, 85°42'W).

####### Paratypes.

CHIOC 33753 a–b.

####### Remarks.

Holotype deposited in CNHE. Other paratypes deposited in CNHE and USNPC.

####### Reference.

Marques et al. (1997a).

###### Acanthobothrium franus

Taxon classificationAnimaliaOnchoproteocephalideaOncobothriidae

Marques, Centritto & Stewart, 1997

####### Type host.

*Narcine entemedor*

####### Infection site.

Spiral valve.

####### Type locality.

Costa Rica, Guanacaste, Gulf of Santa Helena, Cuajiniquil beach (10°57'N, 85°42'W).

####### Paratypes.

CHIOC 33754 a–b.

####### Remarks.

Holotype deposited in CNHE. Other paratypes deposited in CNHE and USNPC.

####### Reference.

Marques et al. (1997a).

###### Acanthobothrium minusculus

Taxon classificationAnimaliaOnchoproteocephalideaOncobothriidae

Marques, Brooks & Barriga, 1997

####### Type host.

*Urobatis tumbesensis* (Chirichigno & McEachran, 1979) [= *Urolophus tumbesensis*] (Chondrichthyes: Urotrygonidae).

####### Infection site.

Anteriormost chamber of spiral valve.

####### Type locality.

Ecuador, Puerto Hualtaco, El Oro (3°16'S, 80°19'W).

####### Paratype.

CHIOC 33693.

####### Remarks.

CHIOC number was not cited in the original description, but the slide deposited by the authors is labeled as a paratype. Holotype deposited in the MEPN collection. Other paratypes deposited in HWML and MHNG.

####### Reference.

Marques et al. (1997b).

###### Order Proteocephalidea

Taxon classificationAnimaliaOnchoproteocephalideaOncobothriidae

Mola, 1928

####### Remarks.

Caira et al. (2014) erected Onchoproteocephalidea containing part of Tetraphyllidea and all Proteocephalidea, which is not accepted by de Chambrier et al (2015). These authors split the genera into subfamilies, without mentioning Monticelliidae and Proteocephalidae. In the present catalogue, these families are maintained, in accordance with the Global Cestode Database (2016).

### Monticelliidae La Rue, 1911

#### *Amazotaenia* de Chambrier, 2001

##### * Amazotaenia yvettae

Taxon classificationAnimaliaOnchoproteocephalideaMonticelliidae

de Chambrier, 2001

###### Type host.

*Brachyplatystoma filamentosum* (Lichtenstein, 1819) (Osteichthyes: Pimelodidae).

###### Infection site.

Intestine.

###### Type locality.

Brazil, Amazonas State, Amazonas River, Itacoatiara.

###### Holotype.

CHIOC 34363.

###### Remarks.

Paratypes deposited in the collections of BMNH and MHNG.

###### Reference.

de Chambrier (2001).

### *Cangatiella* Pavanelli & Santo, 1990

#### * Cangatiella arandasi

Taxon classificationAnimaliaOnchoproteocephalideaMonticelliidae

Pavanelli & Santos, 1990

##### Type host.

*Trachelyopterus galeatus* (Linnaeus, 1766) [= *Parauchenipterus galeatus*] (Osteichthyes: Auchenipteridae).

##### Infection site.

Small intestine.

##### Type locality.

Brazil, Paraná State, Porto Rico.

##### Holotype.

CHIOC 32620 a.

##### Remarks.

No paratype deposited, only vouchers.

##### Reference.

Pavanelli and Santos (1990).

### *Chambriella* Rego, Chubb & Pavanelli, 1999

#### * Chambriella agostinhoi

Taxon classificationAnimaliaOnchoproteocephalideaMonticelliidae

(Pavanelli & Santos, 1992) Rego, Chubb & Pavanelli, 1999

##### Type host.

*Zungaro zungaro* (Humboldt, 1821) [= *Paulicea luetkeni* (Steindachner, 1801)] (Osteichthyes: Pimelodidae).

##### Infection site.

Intestine.

##### Type locality.

Brazil, Paraná State, Paraná River.

##### Holotype.

CHIOC 32820 a.

##### Paratypes.

CHIOC 32820 b–d.

##### Remarks.

Described as *Goezeella agostinhoi* by Pavanelli and Santos (1992).

##### References.

Pavanelli and Santos (1992), Rego et al. (1999).

#### Chambriella paranaensis

Taxon classificationAnimaliaOnchoproteocephalideaMonticelliidae

(Pavanelli & Rego, 1989) Rego, Chubb & Pavanelli, 1999

##### Type host.

*Hemisorubim platyrhynchos*

##### Infection site.

Intestine.

##### Type locality.

Brazil, Paraná State, reservoir of Itaipu, Paraná River, Porto Rico.

##### Holotype.

CHIOC 32490.

##### Paratypes.

CHIOC 32491 a–b.

##### Remarks.

Species described as *Goezeella paranaensis* by Pavanelli and Rego (1989). Types not indicated in the original description by the authors. That information is only in the cataloguing data of CHIOC.

##### References.

Pavanelli and Rego (1989), Rego et al. (1999).

### *Endorchis* Woodland, 1934

#### Endorchis auchenipteri

Taxon classificationAnimaliaOnchoproteocephalideaMonticelliidae

de Chambrier & Vaucher, 1999

##### Type host.

*Auchenipterus osteomystax* (Miranda-Ribeiro, 1918) (Osteichthyes: Auchenipteridae).

##### Infection site.

Intestine.

##### Type locality.

Paraguay, Ñeembucú Department, Paraná River, General E. Diaz.

##### Paratype.

CHIOC 34979.

##### Remarks.

Paratype from CHIOC donated by de Chambrier. Holotype and paratypes deposited in the MHNG collection.

##### Reference.

de Chambrier and Vaucher (1999).

### *Goezeella* Furhmann, 1916

#### Goezeella nupeliensis

Taxon classificationAnimaliaOnchoproteocephalideaMonticelliidae

Pavanelli & Rego, 1991

(= Manaosia bracodemoca Woodland, 1935)

##### Type host.

*Sorubim lima* (Bloch & Schneider, 1801) (Osteichthyes: Pimelodidae).

##### Infection site.

Intestine.

##### Type locality.

Brazil, Paraná State, reservoir of Itaipu, Paraná River, Porto Rico.

##### Holotype.

CHIOC 32562 a.

##### Paratypes.

CHIOC 32562 b–c.

##### Remarks.

de Chambrier and Vaucher (1999) synonymized *Goezella nupeliensis* with *Paramonticellia itaipuensis*. Later, de Chambrier (2003) synonymized *Paramonticellia* with *Manaosia*, and *P. itaipuensis* with *M. bracodemoca*.

##### References.

Pavanelli and Rego (1991), de Chambrier and Vaucher (1999), de Chambrier (2003).

### *Jauella* Rego & Pavanelli, 1985

#### * Jauella glandicephalus

Taxon classificationAnimaliaOnchoproteocephalideaMonticelliidae

Rego & Pavanelli, 1985

##### Type host.

*Zungaro zungaro* (= *Paulicea luetkeni*)

##### Infection site.

Anterior intestine.

##### Type locality.

Brazil, Paraná State, reservoir of Itaipu.

##### Holotype.

CHIOC 32179.

##### Paratype.

CHIOC 32180.

##### Remarks.

CHIOC 32180 was indicated as a paratype in the cataloguing data of CHIOC, but not in the original description. After reviewing the information and slides of *J. glandicephalus*, we concluded that the specimen is a paratype.

##### Reference.

Rego and Pavanelli (1985).

### *Mariauxiella* de Chambrier & Rego, 1995

#### * Mariauxiella pimelodi

Taxon classificationAnimaliaOnchoproteocephalideaMonticelliidae

de Chambrier & Rego, 1995

##### Type host.

*Pimelodus* sp. (Osteichthyes: Pimelodidae).

##### Infection site.

Intestine.

##### Type locality.

Brazil, Mato Grosso State, Cuiabá River, Barão de Melgaço.

##### Paratypes.

CHIOC 33111 a–b, 33112.

##### Remarks.

Holotype and other paratypes deposited in the MHNG collection.

##### Reference.

de Chambrier and Rego (1995).

### *Monticellia* La Rue, 1911

#### Monticellia belavistensis

Taxon classificationAnimaliaOnchoproteocephalideaMonticelliidae

Pavanelli, Machado, Takemoto & Santos, 1994

##### Type host.

*Pterodoras granulosus* (Valenciennes, 1821) (Osteichthyes: Doradidae).

##### Infection site.

Intestine.

##### Type locality.

Brazil, Paraná State, Paraná River, Foz do Iguaçu, reservoir of Itaipu.

##### Holotype.

CHIOC 33122 a.

##### Paratypes.

CHIOC 33122 b–e.

##### Reference.

Pavanelli et al. (1994).

#### Monticellia loyolai

Taxon classificationAnimaliaOnchoproteocephalideaMonticelliidae

Pavanelli & Santos, 1992

(= Monticellia magna (Rego, Santos & Silva, 1974) de Chambrier & Vaucher, 1999 ?

##### Type host.

*Pimelodus maculatus* Lacépède, 1803

##### Infection site.

Intestine.

##### Type locality.

Brazil, Paraná State, Paraná River.

##### Holotype.

CHIOC 32715.

##### Paratypes.

CHIOC 32716 a–d, 32717 a–d.

##### Remarks.

Species synonymized with *M. magna* by de Chambrier and Vaucher (1999), but referred to as “*M. magna* ?” in the Global Cestode Database (2016). *Monticellia magna* was described originally as *Nomimoscolex magna* by Rego et al. (1974).

##### References.

Rego et al. (1974), Pavanelli and Santos (1992), de Chambrier and Vaucher (1999), Global Cestode Database (2016).

### *Nomimoscolex* Woodland, 1934

#### Nomimoscolex chubbi

Taxon classificationAnimaliaOnchoproteocephalideaMonticelliidae

(Pavanelli & Takemoto, 1995) de Chambrier & Vaucher, 1997

##### Type host.

*Gymnotus carapo* (Linnaeus, 1758) (Osteichthyes: Gymnotidae).

##### Infection site.

Anterior intestine diverticles.

##### Type locality.

Brazil, Paraná State, Paraná River, Porto Rico.

##### Holotype.

CHIOC 33188.

##### Paratypes.

CHIOC 33189 a–b.

##### Remarks.

Species described as *Proteocephalus chubbi* by Pavanelli and Takemoto (1995).

##### References.

Pavanelli and Takemoto (1995), de Chambrier and Vaucher (1997).

#### Nomimoscolex lopesi

Taxon classificationAnimaliaOnchoproteocephalideaMonticelliidae

Rego, 1989

##### Type host.

*Pseudoplatystoma fasciatum* (Linnaeus, 1766) (Osteichthyes: Pimelodidae).

##### Infection site.

Intestine.

##### Type locality.

Brazil, Mato Grosso State, Cuiabá River.

##### Holotype.

CHIOC 32553 a.

##### Paratypes.

CHIOC 32553 b–c.

##### Reference.

Rego (1989).

#### Nomimoscolex matogrossensis

Taxon classificationAnimaliaOnchoproteocephalideaMonticelliidae

Rego & Pavanelli, 1990

##### Type host.

*Hoplias malabaricus* (Bloch, 1794) (Osteichthyes: Erythrinidae).

##### Infection site.

Intestine.

##### Type locality.

Brazil, Mato Grosso State, Salobra.

##### Holotype.

CHIOC 32512.

##### Paratype.

CHIOC 32513.

##### Reference.

Rego and Pavanelli (1990).

#### Nomimoscolex pertierrae

Taxon classificationAnimaliaOnchoproteocephalideaMonticelliidae

de Chambrier, Takemoto & Pavanelli, 2006

##### Type host.

*Pseudoplatystoma corruscans* (Spix & Agassiz, 1829)

##### Infection site.

Small intestine.

##### Type locality.

Brazil, Mato Grosso do Sul State, Guaraná Lake (22°39'S, 53°19'W).

##### Holotype.

CHIOC 36598.

##### Paratypes.

CHIOC 36599, 36600 a–b, 32713, 32714.

##### Remarks.

CHIOC 36600 a–b collected in the Paraná River (Porto Rico, Paraná State), and 32713–32714, referred to as *Nomimoscolex sudobim* Woodland, 1935 by Pavanelli and Rego (1992), collected in the Itaipu reservoir (Paraná State). Other paratypes deposited in the BMNH and MHNG collections.

##### References.

Pavanelli and Rego (1992), de Chambrier et al. (2006).

#### Nomimoscolex suspectus

Taxon classificationAnimaliaOnchoproteocephalideaMonticelliidae

Zehnder, de Chambrier, Vaucher & Mariaux, 2000

##### Type host.

*Brachyplatystoma filamentosum*

##### Infection site.

Posterior part of intestine.

##### Type locality.

Brazil, Amazonas State, Itacoatiara.

##### Holotype.

CHIOC 34212 a.

##### Paratypes.

CHIOC 34212 b–c.

##### Remarks.

Other paratypes deposited in the MHNG collection.

##### Reference.

Zehnder et al. (2000).

### *Nupelia* Pavanelli & Rego, 1991

#### * Nupelia portoriquensis

Taxon classificationAnimaliaOnchoproteocephalideaMonticelliidae

Pavanelli & Rego, 1991

##### Type host.

*Sorubim lima*

##### Infection site.

Intestine.

##### Type locality.

Brazil, Paraná State, reservoir of Itaipu, Paraná River, Porto Rico.

##### Holotype.

CHIOC 32558 a.

##### Paratypes.

CHIOC 32558 b–d, 32559 a–b.

##### Remarks.

The slides CHIOC 32558 b–d and 32559 a–b are labeled as paratypes, but the authors did not refer to them in the original description.

##### Reference.

Pavanelli and Rego (1991).

### *Paramonticellia* Pavanelli & Rego, 1991 (= *Manaosia* Woodland, 1935)

#### * Paramonticellia itaipuensis

Taxon classificationAnimaliaOnchoproteocephalideaMonticelliidae

Pavanelli & Rego, 1991

(= Manaosia bracodemoca Woodland, 1935)

##### Type host.

*Sorubim lima*

##### Infection site.

Intestine.

##### Type locality.

Brazil, Paraná State, reservoir of Itaipu, Paraná River, Porto Rico.

##### Holotype.

CHIOC 32560 a.

##### Paratypes.

CHIOC 32560 b–e.

##### Remarks.

CHIOC 32560 d–e are labeled as paratypes, but they were not referred to in the original description. de Chambrier and Vaucher (1999) synonymized *Goezella nupeliensis* with *P. itaipuensis*. Later, de Chambrier (2003) synonymized *Paramonticellia* with *Manaosia*, and *P. itaipuensis* with *M. bracodemoca*.

##### References.

Pavanelli and Rego (1991), de Chambrier and Vaucher (1999), de Chambrier (2003).

### *Pseudocrepidobothrium* Rego & Ivanov, 2001

#### * Pseudocrepidobothrium eirasi

Taxon classificationAnimaliaOnchoproteocephalideaMonticelliidae

(Rego & de Chambrier, 1995) Rego & Ivanov, 2001

##### Type host.

*Phractocephalus hemioliopterus* (Bloch & Schneider, 1801) (Osteichthyes: Pimelodidae).

##### Infection site.

Intestine.

##### Type locality.

Brazil, Amazonas State, Amazonas River, Itacoatiara.

##### Paratype.

CHIOC 33128.

##### Remarks.

Holotype deposited in the MHNG collection. Other paratypes deposited in the BMNH and MHNG. Species described as *Crepidobothrium eirasi* by Rego and de Chambier (1995).

##### References.

Rego and de Chambier (1995), Rego and Ivanov (2001).

### *Spassklyellina* Freze, 1965

#### Spassklyellina mandi

Taxon classificationAnimaliaOnchoproteocephalideaMonticelliidae

Pavanelli & Takemoto, 1996

##### Type host.

*Pimelodus ornatus* Kner, 1858

##### Infection site.

Intestine.

##### Type locality.

Brazil, Paraná State, Paraná River, Porto Rico.

##### Holotype.

CHIOC 33679 a.

##### Paratypes.

CHIOC 33679 b–d

##### Reference.

Pavanelli and Takemoto (1996).

### *Spatulifer* Woodland, 1934

#### Spatulifer maringaensis

Taxon classificationAnimaliaOnchoproteocephalideaMonticelliidae

Pavanelli & Rego, 1989

##### Type host.

*Hemisorubim platyrhynchos*

##### Infection site.

Intestine.

##### Type locality.

Brazil, Paraná State, reservoir of Itaipu, Paraná River, Porto Rico.

##### Holotype.

CHIOC 32487.

##### Paratypes.

CHIOC 32488 a–c.

##### Remarks.

Types not indicated in the original description by the authors. That information is in the cataloguing data of CHIOC.

##### Reference.

Pavanelli and Rego (1989).

### *Vaucheriella* de Chambrier, 1987

#### * Vaucheriella bicheti

Taxon classificationAnimaliaOnchoproteocephalideaMonticelliidae

de Chambrier, 1987

##### Type host.

Tropidophis cf. taczanowskyi (Steindachner, 1880) (Serpentes: Tropidophiidae).

##### Infection site.

Intestine.

##### Type locality.

Ecuador, Zamora-Chinchipe.

##### Paratype.

CHIOC 33025.

##### Remarks.

Holotype and paratypes deposited in the MHNG collection. Paratype from CHIOC donated by de Chambrier. Other paratypes deposited in BMNH and MNHN.

##### Reference.

de Chambrier (1987).

### Proteocephalidae La Rue, 1911

#### *Euzetiella* de Chambrier, Rego & Vaucher, 1999

##### * Euzetiella tetraphylliformis

Taxon classificationAnimaliaOnchoproteocephalideaProteocephalidae

de Chambrier, Rego & Vaucher, 1999

###### Type host.

*Zungaro zungaro* (= *Paulicea luetkeni*)

###### Infection site.

Anterior intestine.

###### Type locality.

Brazil, Amazonas State, Itacoatiara.

###### Paratypes.

CHIOC 33940 b–c.

###### Remarks.

Holotype and other paratypes deposited in the MHNG collection.

###### Reference.

de Chambrier et al. (1999).

### *Frezeella* Alves, de Chambrier, Scholz & Luque, 2015

#### * Frezeella vaucheri

Taxon classificationAnimaliaOnchoproteocephalideaProteocephalidae

Alves, de Chambrier, Scholz & Luque, 2015

##### Type host.

*Tocantinsia piresi* (Miranda-Ribeiro, 1920) (Osteichthyes: Auchenipteridae).

##### Infection site.

Anterior intestine.

##### Type locality.

Brazil, Pará State, Altamira, Xingú River (3°12'S, 52°12'W).

##### Holotype.

CHIOC 37978 a–h (complete specimen and cross sections on eight slides).

##### Paratypes.

CHIOC 37979 a–d, 37980 a–d.

##### Remarks.

Other paratypes deposited in the IPCAS and MHNG collections.

##### Reference.

Alves et al. (2015).

### *Megathylacus* Woodland, 1934

#### Megathylacus brooksi

Taxon classificationAnimaliaOnchoproteocephalideaProteocephalidae

Rego & Pavanelli, 1985

##### Type host.

*Zungaro zungaro* (= *Paulicea luetkeni*)

##### Infection site.

Anterior intestine.

##### Type locality.

Brazil, Paraná State, reservoir of Itaipu.

##### Holotype.

CHIOC 32184.

##### Paratype.

CHIOC 32183.

##### Remarks.

CHIOC 32183 was indicated as a paratype in the cataloguing data of CHIOC, but not in the original description. After reviewing the information and slides of *M. brooksi*, we concluded that the specimen is a paratype. Collection numbers are referred to as “29183” and “29184” in the original description due to a mistake. *Megathylacus brooksi* is considered conspecific with *M. jandia* Woodland, 1934 by de Chambrier et al. (2014), but is considered a valid name in the Global Cestode Database (2016).

##### References.

Rego and Pavanelli (1985), de Chambrier et al. (2014), Global Cestode Database (2016).

#### Megathylacus travassosi

Taxon classificationAnimaliaOnchoproteocephalideaProteocephalidae

Pavanelli & Rego, 1992

##### Type host.

*Pseudoplatystoma corruscans*

##### Infection site.

Intestine.

##### Type locality.

Brazil, Paraná State, Paraná River.

##### Holotype.

CHIOC 32710.

##### Remarks.

No paratypes deposited, only vouchers.

##### Reference.

Pavanelli and Rego (1992).

### *Proteocephalus* Weinland, 1858

#### Proteocephalus gibsoni

Taxon classificationAnimaliaOnchoproteocephalideaProteocephalidae

Rego & Pavanelli, 1991

##### Type host.

*Astronotus ocellatus* (Agassiz, 1831) (Osteichthyes: Cichlidae).

##### Infection site.

Intestine.

##### Type locality.

Brazil, Amazonas State, Manaus, Amazonas River.

##### Holotype.

CHIOC 32508.

##### Paratypes.

CHIOC 32509 a–b.

##### Remarks.

Paratypes collected in Maicurú (Pará State) from *Astronotus* sp. Species referred to as *P. ocellatus* by Rego and Pavanelli (1990) and renamed by Rego and Pavanelli (1991) because the original name had already been used.

##### References.

Rego and Pavanelli (1990), Rego and Pavanelli (1991).

#### Proteocephalus hemioliopteri

Taxon classificationAnimaliaOnchoproteocephalideaProteocephalidae

(Rego, 1984) de Chambrier & Vaucher, 1997

##### Type host.

*Phractocephalus hemioliopterus* (= *Phractocephalus hemiliopterus*)

##### Infection site.

Intestine.

##### Type locality.

Brazil, Amazonas State, Amazonas River.

##### Syntypes.

CHIOC 32088 a–b, d–e, g.

##### Remarks.

Species referred to as *Myzophorus woodlandi* by Rego (1984) and *Nomimoscolex woodlandi* by Rego and Pavanelli (1992). de Chambrier and Vaucher (1997) proposed a new name, *Proteocephalus hemioliopteri*, for *N. woodlandi* to avoid homonymy with *P. woodlandi* Moghe, 1926. Rego (1984) did not designate holotype and paratypes or labeled the slides as type material. Among the syntypes referred to as “32088 a–g” in the original description, the specimens “c” and “f” were analyzed, separated and reallocated by de Chambrier in CHIOC, because they belong to another genus.

##### References.

Rego (1984), Rego and Pavanelli (1992), de Chambrier and Vaucher (1997).

#### Proteocephalus hobergi

Taxon classificationAnimaliaOnchoproteocephalideaProteocephalidae

de Chambrier & Vaucher, 1999

##### Type host.

*Oxydoras kneri* Bleeker, 1862 (Osteichthyes: Doradidae).

##### Infection site.

Intestine.

##### Type locality.

Paraguay, Ñeembucú Department, 5 km Northwest of Pilar.

##### Paratype.

CHIOC 34978.

##### Remarks.

Paratype from CHIOC donated by de Chambrier. It was collected in the Paraguay River, San Antonio, Central Department (Paraguay). Holotype and paratypes deposited in the MHNG collection.

##### Reference.

de Chambrier and Vaucher (1999).

#### Proteocephalus joanae

Taxon classificationAnimaliaOnchoproteocephalideaProteocephalidae

de Chambrier & Paulino, 1997

##### Type host.

*Xenodon neuwiedii* Günther, 1863 [= *Xenodon neuwiedi*] (Serpentes: Colubridae).

##### Infection site.

Anterior part of intestine.

##### Type locality.

Brazil, São Paulo State, Pariquera-açu.

##### Paratypes.

CHIOC 33537, 33745 a–c.

##### Remarks.

Paratypes from CHIOC collected in Mogi das Cruzes (São Paulo State). Holotype deposited in the MHNG collection. Other paratypes deposited in BMNH, IPCAS and MHNG.

##### Reference.

de Chambrier and Paulino (1997).

#### Proteocephalus renaudi

Taxon classificationAnimaliaOnchoproteocephalideaProteocephalidae

de Chambrier & Vaucher, 1994

##### Type host.

*Platydoras costatus* (Linnaeus, 1758) (Osteichthyes: Doradidae).

##### Infection site.

Intestine.

##### Type locality.

Paraguay, Ñeembucú Department, General E. Diaz.

##### Paratypes.

CHIOC 32888, 34958.

##### Remarks.

Holotype deposited in the MHNG collection. Other paratypes deposited in BMNH and MHNG.

##### Reference.

de Chambrier and Vaucher (1994).

#### Proteocephalus serrasalmus

Taxon classificationAnimaliaOnchoproteocephalideaProteocephalidae

Rego & Pavanelli, 1990

##### Type host.

*Serrasalmus spilopleura* Kner, 1858 (Osteichthyes: Serrasalmidae).

##### Infection site.

Intestine.

##### Type locality.

Brazil, Paraná State, Paraná River.

##### Holotype.

CHIOC 32505.

##### Paratype.

CHIOC 32506.

##### Reference.

Rego and Pavanelli (1990).

#### Proteocephalus sophiae

Taxon classificationAnimaliaOnchoproteocephalideaProteocephalidae

de Chambrier & Rego, 1994

##### Type host.

*Zungaro zungaro* (= *Paulicea luetkeni*)

##### Infection site.

Anterior intestine diverticles.

##### Type locality.

Brazil, Amazonas State, Amazonas River, Itacoatiara.

##### Paratype.

CHIOC 33114.

##### Remarks.

Holotype and other paratypes deposited in the MHNG collection.

##### Reference.

de Chambrier and Rego (1994).

#### Proteocephalus vazzolerae

Taxon classificationAnimaliaOnchoproteocephalideaProteocephalidae

Pavanelli & Takemoto, 1995

##### Type host.

*Piaractus mesopotamicus* (Holmberg, 1887) (Osteichthyes: Serrasalmidae).

##### Infection site.

Anterior intestine diverticles.

##### Type locality.

Brazil, Paraná State, Paraná River, Porto Rico.

##### Holotype.

CHIOC 33187 a.

##### Paratype.

CHIOC 33187 b.

##### Reference.

Pavanelli and Takemoto (1995).

### *Travassiella* Rego & Pavanelli, 1987

#### * Travassiella avitellina

Taxon classificationAnimaliaOnchoproteocephalideaProteocephalidae

Rego & Pavanelli, 1987

##### Type host.

*Zungaro zungaro* (= *Paulicea luetkeni*)

##### Infection site.

Median intestine.

##### Type locality.

Brazil, Mato Grosso State, Salobra.

##### Holotype.

CHIOC 32259.

##### Remarks.

Species synonymized with *Proteocephalus jandia* Woodland, 1934, which was transferred to *Travassiella* Rego & Pavanelli, 1987 as *Travassiella jandia* (Woodland, 1934) n. comb. by de Chambrier et al. (2014). However, *T. avitellina* is accepted in the Global Cestode Database (2016).

##### References.

Rego and Pavanelli (1987), de Chambrier et al. (2014), Global Cestode Database (2016).

### Order Rhinebothriidea Healy, Caira, Jensen, Webster & Littlewood, 2009

#### Anthocephaliidae Ruhkne, Caira & Cox, 2015

##### *Anindobothrium* Marques, Brooks & Lasso, 2001

###### Anindobothrium lisae

Taxon classificationAnimaliaRhinebothriideaAnthocephaliidae

Marques, Brooks & Lasso, 2001

####### Type host.

*Potamotrygon orbignyi* (Castelnau, 1855) (Chondricthyes: Potamotrygonidae).

####### Infection site.

Spiral valve.

####### Type locality.

Brazil, Amazonas State, Rio Negro near Barcelos (00°59'S, 62°58'W).

####### Holotype.

CHIOC 34375.

####### Remarks.

Paratypes deposited in HWML, INPA and USNPC.

####### Reference.

Marques et al. (2001).

### Rhinebothriidae Euzet, 1953

#### *Rhinebothroides* Mayes, Brooks & Thorson, 1981

##### Rhinebothroides mclennanae

Taxon classificationAnimaliaRhinebothriideaRhinebothriidae

Brooks & Amato, 1992

(= R. glandularis Brooks, Mayes & Thorson, 1981)

###### Type host.

*Potamotrygon motoro* (Müller & Henle, 1841)

###### Infection site.

Middle third of spiral valve.

###### Type locality.

Brazil, Mato Grosso do Sul State, Corumbá.

###### Holotype.

CHIOC 32814 a.

###### Paratypes.

CHIOC 32814 b–f.

###### Remarks.

Other paratypes deposited in the HWML collection. Species synonymized with *R. glandularis* by Marques and Brooks (2003).

###### References.

Brooks and Amato (1992), Marques and Brooks (2003).

### Order Trypanorhyncha Diesing, 1863

#### Eutetrarhynchidae Guiart, 1927

##### *Dollfusiella* Campbell & Beverige, 1994

###### Dollfusiella vooremi

Taxon classificationAnimaliaTrypanorhynchaEutetrarhynchidae

(São Clemente & Gomes, 1989) Beveridge, Neifar & Euzet, 2004

####### Type host.

*Mustelus canis* (Mitchill, 1815) (Chondrichthyes: Triakidae).

####### Infection site.

Spiral valve.

####### Type locality.

Brazil, off Rio Grande do Sul State (33°40'S, 50°40'W).

####### Holotype.

CHIOC 32566 e.

####### Paratypes.

CHIOC 32566 a–d.

####### Remarks.

CHIOC 32566 c collected from *Mustelus schmitti* Springer, 1939. Species originally described as *Eutetrarhynchus vooremi* by São Clemente and Gomes (1989).

####### References.

São Clemente and Gomes (1989), Beveridge et al. (2004).

#### Lacistorhynchidae Guiart, 1927

##### Grillotia (Christianella) Guiart, 1931

###### Grillotia (Christianella) carvajalregorum

Taxon classificationAnimaliaTrypanorhynchaLacistorhynchidae

(Carvajal & Rego, 1983) Menoret & Ivanov, 2009

####### Type host.

*Cynoscion striatus* (Cuvier, 1829) (Osteichthyes: Sciaenidae).

####### Infection site.

Visceral cavity.

####### Type locality.

Brazil, Rio de Janeiro State, Rio de Janeiro, Fish market.

####### Holotype.

CHIOC 32018 a.

####### Paratypes.

CHIOC 32018 b–d.

####### Remarks.

Other paratypes deposited in the collection of the Department of Zoology, University of Concepción, Chile. Species originally described as *Progrillotia dollfusi* by Carvajal and Rego (1983).

####### References.

Carvajal and Rego (1983), Menoret and Ivanov (2009).

##### Tentaculariidae Poche, 1926

###### *Heteronybelinia* Palm, 1999

####### Heteronybelinia annakohnae

Taxon classificationAnimaliaTrypanorhynchaTentaculariidae

Pereira Jr. & Boeger, 2005

######## Type host.

*Ctenosciaena gracilicirrhus* (Metzellar, 1919) (Osteichthyes: Sciaenidae).

######## Infection site.

Coelomic cavity.

######## Type locality.

Brazil, Rio Grande do Sul State, Rio Grande.

######## Holotype.

CHIOC 33790.

######## Paratypes.

CHIOC 33791, 33792, 33793, 33794.

######## Remarks.

Paratypes collected from other sciaenid fish: CHIOC 33791 from *Micropogonias furnieri*; 33792 and 33793 from *Cynoscion guatucupa* (Cuvier, 1830); 33794 from *Cynoscion jamaicensis* (Vaillant & Bocourt, 1883); and 33795 from *Menticirrhus americanus* (Linnaeus, 1758). Other paratypes deposited in USNPC.

######## Reference.

Pereira Jr. and Boeger (2005).
